# COVID-19 mobility restrictions and stay-at-home behaviour in 2020 were associated with higher retail food prices worldwide

**DOI:** 10.1016/j.gfs.2023.100702

**Published:** 2023-06

**Authors:** Jessica Wallingford, Elena M. Martinez, William A. Masters

**Affiliations:** a Friedman School of Nutrition Science and Policy, Tufts University, Boston, MA, USA; b Department of Economics, Tufts University, Medford, MA, USA

**Keywords:** Pandemic response, Food security, Diet costs, Affordability

## Abstract

COVID-19 policy responses have included mobility restrictions, and many people have chosen to stay at home to avoid exposure. These actions have ambiguous impacts on food prices, lowering demand for food away from home and perishables, while increasing supply costs for items where workers are most affected by the pandemic. We use evidence from 160 countries to identify the net direction and magnitude of association between countries' real cost of all food and mobility restriction stringency. We investigate the deviation of each month's price level in 2020 from that month's average price level during the previous three years and find that an increase in mobility restriction stringency from no restrictions to most restrictive is associated with an increase in the real cost of all food of more than one percentage point across all models. We then examine the relationship between retail food price levels by food group and stay-at-home behaviour around markets in 36 countries and find positive associations for non-perishables, dairy and eggs.

## Highlights

• Worldwide retail prices rose more for food than all consumer goods during COVID.
• We find larger food price rises at times and places with more stringent restrictions.
• For non-perishables we find prices were higher when more people stayed home.
• For fruits and vegetables we find prices were lower when more people stayed home.
• New retail price data can help track response to shocks in global food systems.

## 1 Introduction

COVID-19 disrupted economies and health systems across the globe, stressing agri-food supply chains in ways that threaten access to affordable diets worldwide. Global monitoring and response has focused on the supply of agricultural commodities traded on international markets (AMIS, 2022; FAO 2022a, FAO-IFAD-the World Bank-WFP, 2020; WCO-WTO 2020), while individual country studies have focused on conditions in specific places that may not be representative of the global population (Pandey et al., 2022; Rudin-Rush et al., 2022; Tabe-Ojong et al., 2022). These analyses highlighted the importance of safety nets and continued cross-border flows from areas of surplus to areas of deficit. Monitoring the prices of raw agricultural commodities remains crucial to world food security, but does not track changes in supply-demand balance for distribution and retailing of retail items needed by individuals and households within countries. This study provides a first effort at global analysis of retail food prices linked to COVID-19 responses, demonstrating the value and feasibility of monitoring world food prices at the consumer level.

Focusing on consumer prices of retail food items, instead of the traditional focus on prices of farm commodities, is particularly important now due to disease impacts on consumers and food system workers in processing and distribution. Policies and behavioral changes designed to restrict transmission could raise costs along the supply chain in tasks that involve face-to-face contact among workers, while lowering demand for retail items and services that involve face-to-face contact with consumers.

To quantify the nature and magnitude of policy restrictions we use the Oxford COVID-19 Government Response Tracker (OxCGRT) (Hale et al., 2021), which is available monthly on a national-average basis for over 180 countries worldwide. Individuals’ behavioral response can precede or follow these policy actions and will differ by type of food. To capture individual choices we apply an admin 1-level index of stay-at-home behavior from the Google Community Mobility Trends data previously used in other studies of COVID-19 response in Africa (Milusheva et al., 2021), matched to location-specific prices for items in each nutritional food group across 36 low- and middle-income countries.

Governments' mobility restrictions and individuals’ stay-at-home behaviour, as well as the disease itself, could affect supply chains and retail prices through impact on workers or through consumer demand for different types of food (Fig. S1). Some disruptions are inherently temporary, for example repackaging for individual sale of items that had previously been intended for distribution to food service providers (Malone et al., 2021; Hobbs 2020; Yaffe-Bellany and Corkery 2020). More sustained impacts include shifts in demand between types of food, and supply chain cost increases due to worker absences and social distancing or other accommodations in the workplace (Hobbs 2020; FAO 2020; FERN 2020; Gray 2020; Laborde et al., 2020; Malone et al., 2021; Mahajan and Tomar 2021; Parks et al., 2020; Richards and Rickard 2020).

**Fig. 1:**
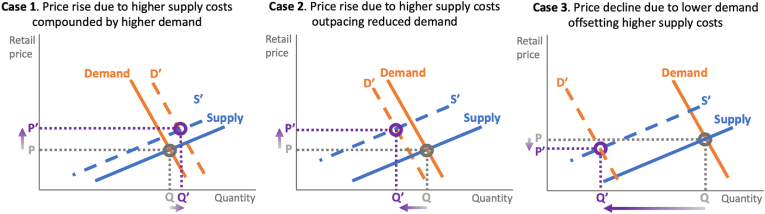
Price change scenarios in response to supply and demand shifts associated with the COVID-19 pandemic. **Notes:** Solid blue and orange lines represent original supply and demand functions, and dashed blue and orange lines represent shifted supply and demand functions. Grey circles represent the initial equilibrium between supply and demand, while purple circles represent the new equilibrium following shifts in supply and demand. (For interpretation of the references to colour in this figure legend, the reader is referred to the Web version of this article.)

While the available evidence suggests that food supplies were largely maintained during the period covered in our analysis (Béné et al., 2021), the alterations in supply and demand dynamics that accompany mobility restrictions could potentially translate to systematically higher or lower food prices. Fig. 1 illustrates potential price change scenarios that might occur at the retail stage in response to supply and demand shifts induced by mobility restriction policies and changes in stay-at-home behaviour. For example, shifts in demand away from restaurants and towards grocery stores, combined with higher supply costs, may result in price increases for some foods (see case 1 in Fig. 1). Alternatively, higher supply costs combined with reductions in demand may result in a net rise in price (Fig. 1, case 2) or a net decrease in price (Fig. 1, case 3), depending on the relative magnitudes of supply and demand shifts.

A small number of studies focusing on specific countries or groups of countries has begun to examine the relationship between COVID-19 mobility restriction policies and retail food prices, though none provide a global perspective (Akter 2020; Ruan et al., 2021.; Narayanan and Saha 2021; Agyei et al., 2021; Dietrich et al., 2021). This study offers a global perspective of a global crisis and uses all available data during the year 2020 that match COVID-19 response to food price levels, first for policy restrictions at the national level, and then for behavioral responses around specific food markets. Our national-level analysis refers to each country's overall consumer price index (CPI) for all food and beverage items, in real terms compared to the country's CPI for all items. Those data are compiled for almost all countries of the world by the Food and Agriculture Organization (FAO) of the United Nations (FAO 2022b) and can be matched to the Oxford COVID-19 Government Response Stringency Index data (Hale et al., 2021) for 160 countries. Our market-level analysis uses Google's Community Mobility Trends data as a subnational and more direct measure of stay-at-home behaviour, matched to item prices from international food market information and early warning systems (EWS) at specific market locations in 36 countries.

This study provides a first global assessment of how retail consumer prices differ during periods of more stringent mobility restriction policies, and at places and times with more stay-at-home behavior, when controlling for disease case counts and other factors. Previous work on global retail food prices has shown higher prices associated with higher COVID-19 case counts (Bai et al., 2022). Adding the effects of mobility restriction policies and stay-at-home behavior provides additional insight into how government and individual response to the pandemic might affect food markets. Over the 160 countries for which we have data in our national-level analysis, we find that more stringent government mobility restrictions are associated with higher overall food CPI but not higher price levels for other goods and services, and over the smaller dataset in our market-level analysis we find that more extreme stay-at-home behavior is associated with higher prices for non-perishables that can be stored at home, as well as higher prices for dairy and eggs that were particularly affected by shifts in purchasing behavior away from food service locations and towards retail locations (Malone et al., 2021). Governments that anticipate these price rises could help avoid the resulting hardship by protecting incomes or providing complementary aid, such as nutritional assistance programs, during periods of lockdown that may be needed to limit the spread of infectious disease.

## 2 Data and methodology

### 2.1 Data for national-level analysis

Our national-level analysis combines: 1) national-level monthly CPI data, for all items and for all food and beverage items, 2) national-level monthly mobility restriction policy stringency data, and 3) national-level monthly data on COVID-19 cases and deaths per million. These data are described in detail below.

#### 2.1.1 Consumer price indices for food and beverages versus other goods and services

Our analysis uses monthly national overall consumer price indices (CPI) and its sub-index for food and non-alcoholic beverages (FCPI) for 160 countries from January 2017 to December 2020, as reported by each country's national statistical organization to the United Nations Statistics Division and the International Monetary Fund (IMF, 2021), and compiled by the Food and Agricultural Organization of the UN (FAO 2022b). At the time data were downloaded for use in our analysis (March 17, 2022), the IMF provided more recent data for some countries, but the FAO provided broader geographic coverage as shown in the annex of Supplemental Information Fig. S2. Within the FAO compilation, we used only values reported from international reliable sources and official data, and excluded values marked as FAO estimates. We then used each country's CPI and FCPI to calculate a third index representing the ratio of food and non-alcoholic beverage price levels to all other consumer goods (FCPI/CPI*100), which we refer to as the food price index (FPI). We then calculated deviations of price index values in 2020 from the average pre-COVID-19 2017–2019 price index value in the same country and month.

#### 2.1.2 Stringency of government mobility restriction policies

Data on the stringency of government policies to restrict mobility were obtained on April 28, 2021 from the Oxford COVID-19 Government Response Tracker Stringency Index. That composite metric ranges from 0 (no restriction) to 100 (most restrictive), constructed by researchers in the Blavatnik School of Government from nine sub-indices reflecting 1) School closures; 2) Workplace closures; 3) Cancelled public events; 4) Restrictions on gatherings; 5) Public transport closures; 6) Public information campaigns; 7) Stay-at-home measures; 8) Restrictions on internal movement; and 9) International travel controls (OxCGRT, 2020; Hale et al., 2021). National-level daily stringency scores are provided for each country beginning January 1, 2020, which we rescaled from 0 to 1 and then used to calculated monthly averages covering the time period of CPI data.

#### 2.1.3 COVID-19 cases and mortality

National-level daily new COVID-19 case counts and mortality were obtained on April 28, 2021 from the World Health Organization (WHO 2021). These data were used to calculate COVID-19 cases per million per month and COVID-19 mortality per million per month for each country for which we had CPI and mobility restriction policy stringency data. These data were included as controls to adjust for pandemic severity in each country and month.

### 2.2 Data for market-level analysis

Our market-level analysis combines: 1) monthly retail item price data collected from markets monitored by early warning systems, 2) admin 1-level monthly stay-at-home behavior data, and 3) national-level monthly data on COVID-19 cases and deaths per million. These data are described in detail below.

#### 2.2.1 Retail item prices from early warning systems

Monthly food item price data were obtained from EWS databases published by three different international agencies, including the FAO Global Information and Early Warning System (GIEWS), the Famine Early Warning System Network (FEWS NET) funded by the United States Agency for International Development (USAID), and the Vulnerability Analysis and Mapping (VAM) system from the World Food Programme (WFP). We use monthly retail item price data from markets in 36 countries from January 2020 to December 2020. Each price for each food item in each market and month was normalized to the baseline period of January 2020 to transform the data from local currencies to unitless price levels that can be compared across countries. We use a baseline period of January 2020 in order to align as closely as possible with the pre-COVID-19 baseline period used by Google's COVID-19 Community Mobility Trends data (detailed in section *2.2.2* below). We then calculate month-to-month percentage point change in price level for each food item in each market. Food items were grouped into one of eight categories (breads and cereals; nuts, seeds, and pulses; fruits and vegetables; meat; fish and seafood; dairy and eggs; oils and fats; sugar and confectionary) based on an adapted version of the COICOP classification system, detailed in Bai et al. (2021b, 2022). The proportion of price observations categorized into each food group in each country is provided in Fig. S3.

#### 2.2.2 Stay-at-home behavior measured using movement trends of mobile phone users

To track stay-at-home behavior in response to the COVID-19 pandemic, we use Google's COVID-19 Community Mobility Trends data. These metrics are compiled from Android mobile device users who have their location history turned on. In low- and middle-income countries, people who use Android smartphones for navigation in this way are likely to have travel behavior that is even more responsive to news about disease risks than the general population, as shown for Uganda by Milusheva et al. (2021). Google mobility data shows how individuals who use location data adjust their travel patterns in response to social media, news reports or other influences, making it a particularly useful sentinel observation of disease perceptions. The aggregated data provided by Google provides daily changes in the number of visitors and average time spent in six categories of places (retail & recreation, grocery & pharmacy, parks, transit stations, workplaces, and residential), at three levels of spatial aggregation (national, admin-1, and admin-2), each shown as percent changes in daily mobility relative to a five-week baseline period from 5 January 2020 to 6 February 2020. For our purposes, we use only the residential place category as a catch-all index for consumer activity, given that more time spent at home should reflect an overall lower degree of consumer activity across place categories outside of the home. We refer to this category of mobility as ‘stay-at-home behaviour’, where higher values indicate more time spent in the home. Few EWS market locations could be matched to stay-at-home behavior data aggregated at the admin-2 level, the lowest level of spatial aggregation. In order to allow for a larger sample, we use the degree to which android phone users stay-at-home, aggregated at the admin-1 level and matched to market locations across 36 mostly low- and middle-income countries, then averaged over the calendar month to match with monthly item prices in each food group.

#### 2.2.3 COVID-19 cases and mortality

Daily new COVID-19 case counts and mortality were downloaded from the Our World in Data COVID-19 Data Explorer, which originally sources these data from the COVID-19 Data Repository by the Center for Systems Science and Engineering (CSSE) at Johns Hopkins University and provides better coverage of the countries included in our market-level analysis. COVID-19 case counts and mortality data were used to calculate COVID-19 cases per million per month and COVID-19 mortality per million per month for each country and were matched to market locations with EWS retail food item price data.

### 2.3 Geographical coverage

**Fig. 2:**
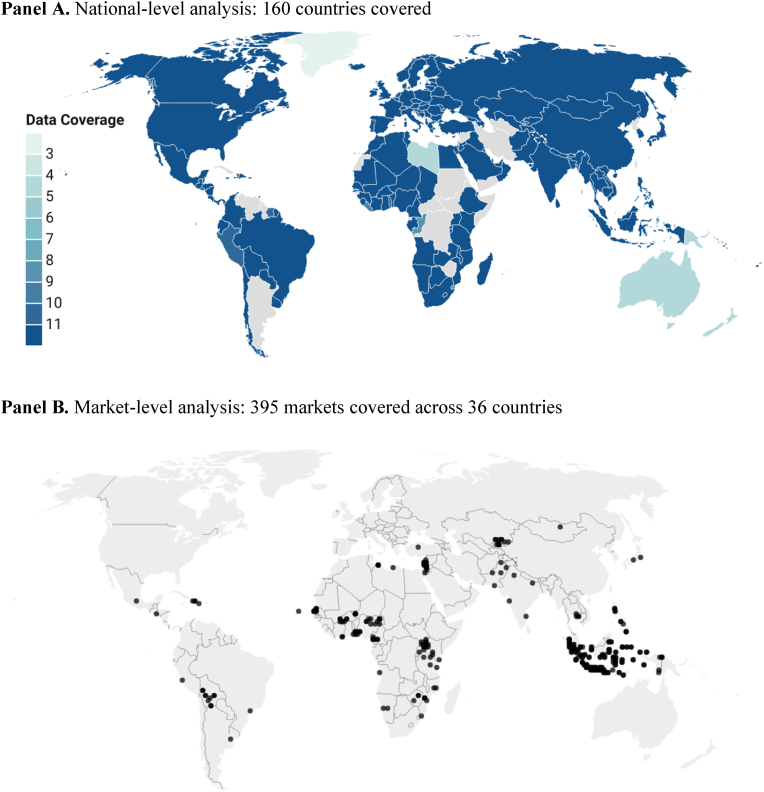
Geographical coverage of price data, 2020 **Notes:** Panel A shows the 160 countries included in our national-level analysis, with darker shades of blue indicating a larger number of months during the year 2020 for which complete price index data, stringency data, and COVID-19 case and mortality data were available. Countries in grey were not included in the national-level analysis. Panel B shows the 395 market locations monitored by early warning systems and included in our market-level analysis.

In our national-level analysis, we compiled all available consumer price index data from the years 2017–2020, COVID-19 mobility restriction stringency data from the year 2020, and COVID-19 case and mortality data from the year 2020. An initial set of 168 countries had consumer price index data, stringency data, and COVID-19 case and mortality data, from which we excluded 8 countries due to anomalous outlier values of consumer price indices. Our exclusion criterion was having one or more observations beyond 15 times the interquartile range. Excluded countries were Argentina, Iran, Lebanon, South Sudan, Sudan, Syria, Venezuela, and Zimbabwe, for which descriptive statistics are provided in Table S1. A final sample of 160 countries was used in our national-level analysis. Among these countries, only months with complete sets of consumer price index data (CPI, FCPI, and FPI) for all years from 2017 through 2020 were retained for analysis. Fig. 2 identifies the 160 countries included in our analysis in blue, with the number of months covered in each country during the year 2020 indicated by the shade of blue. The number of countries covered in each month of the year 2020 is listed in Table S2.

Our market-level analysis includes all EWS monthly retail item price data from the year 2020, in markets that can be matched to admin-1 level stay-at-home behavior data and national-level COVID-19 case and mortality data. We used the winsor2 Stata package to trim the top and bottom 0.5 percent of price observations within each food group category to remove extreme values likely due to data entry errors. This resulted in a final sample of 68,167 price observations from 395 markets, shown in panel B of Fig. 2, from 132 admin 1 units across 36 countries. Table S3 presents the number of price observations in each month beginning in February of 2020 and ending in December of 2020.

### 2.4 Empirical strategy

Stata SE/17.0 was used for statistical analysis and visualizations; Datawrapper was used to create maps.

#### 2.4.1 National-level analysis: mobility restriction stringency and consumer price indices

To measure the relationship between stringency in COVID-19 mobility restrictions and the deviation of each month's food price level from the average 2017–2019 price level in the same country and month, we use OLS regression with the following baseline specification:

(1) $$P_{it}=\beta_{0}+\beta_{1}{stringency}_{it}+\beta_{2}{\ln\left(C19case+1\right)}_{it}+\beta_{3}{\ln\left(C19mortality+1\right)}_{it}+u_{i}+\delta_{it}+\varepsilon_{it}\text{,}$$

In these equations *P* represents the outcome of interest, which is the price level in each country, *i*, and month, *t*, of calendar year 2020 relative to the same month in the three previous years (2017–2019). Data are percentage point changes, to show for example that price levels in June 2020 were 11 percentage points above the average of June 2017, June 2018 and June 2019. We test for price differences using three different price levels, first for all food and beverages in each country (FCPI), then for all goods and services of any type (CPI), and then the real food price level relative to all other things (FPI). Our model includes terms for COVID-19 cases per million and mortality per million as proxies to control for pandemic severity in each country and month. COVID-19 case and mortality data were log(x+1)-transformed to reduce skewedness in their distributions. Our model includes country fixed effects and country time trends, and uses robust standard errors clustered at the country level to account for heteroskedasticity. In alternative specifications, we test the robustness of our results to the inclusion of one period lag terms for mobility restriction stringency, COVID-19 cases, and COVID-19 mortality. All data sources and variables included in our models are summarized in Table S4.

#### 2.4.2 Market-level analysis: stay-at-home behavior and EWS food item prices

To measure the correlation between retail food prices and stay-at-home behavior, we use OLS regression with the following specification for each food item (*j*) at each market location (*i*) and month (*t*):

(2) $$P_{jit}-P_{ji\left(t-1\right)}=\beta_{0}+\beta_{1}\left({StayAtHomeBehavior}_{it}-{StayAtHomeBehavior}_{i\left(t-1\right)}\right)+\beta_{2}{FG}_{j}+\beta_{3}\left({FG}_{j}*\;\left({StayAtHomeBehavior}_{it}-{StayAtHomeBehavior}_{i\left(t-1\right)}\right)\right)+\beta_{4}X_{it}^{\prime }+u_{i}+\theta_{t}+\delta_{it}+\varepsilon_{jit}\text{,}$$

Where *P*_*jit*_ *– P*_*ji(t-*1*)*_ represents the percentage point change in retail price level between month *t* and the previous month, *t-*1, for each food item (*j*) at every market (*i*) for which data are available. The term *StayAtHomeBehavior*_*it*_ - *StayAtHomeBehavior*_*i(t-*1*)*_ represents the percentage point change in stay-at-home behaviour between month *t* and the previous month, *t-*1. *FG*_*j*_ represents a categorical variable that assigns each food item to one of eight different food groups, using breads and cereals as the reference category. The coefficient, β_3_, on the interaction term is the effect of interest and represents the effect of month-to-month change in stay-at-home behaviour on month-to-month change in retail prices for a given food group, compared to the reference change in price level for breads and cereals. *X'*_*it*_ represents a vector of time-varying factors including confirmed new COVID-19 cases and mortality per month per million, which were log(x + 1)-transformed to minimize skewedness in their distributions. We also include fixed effects for each market location, $u_{i}$, to account for unobserved heterogeneity over space, month fixed effects, $\theta_{t}\text{,}$ to account for worldwide shocks in a given month, and country time trends, $\delta_{it}\text{,}$ to account for country-specific trends of price inflation. All models use robust standard errors to account for heteroskedasticity, and are clustered at the market level. In alternative specifications, we examine the robustness of our results to the introduction of month-to-month change in mobility restriction stringency in place of month-to-month change in stay-at-home behavior.

Importantly, the results from our analysis do not adjust for heterogeneity of effects over time and space. Future work should consider issues arising from serial and spatial correlation in unobserved factors that influence our estimated average coefficients, using recent techniques developed for two-way fixed effects and difference-in-difference models that combine multiple kinds of fixed effects, and account for heterogeneity of response to shocks within each group (Baker et al., 2022; De Chaisemartin and D’Haultfœuille, 2022).

## 3 Results

### 3.1 National-level analysis

#### 3.1.1 Global trends in retail price levels for food and other consumer goods

**Fig. 3:**
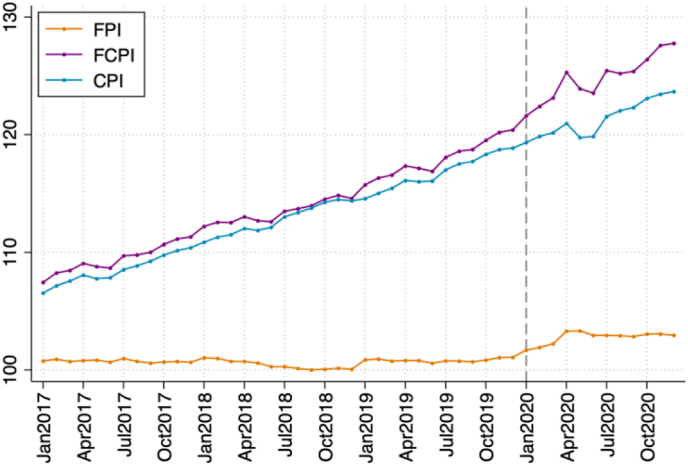
Global average price index levels for food and other consumer goods, January 2017 to December 2020 **Notes**: Consumer price index data were obtained from the Food and Agriculture Organization of the United Nations on March 17, 2022 and include each country's national consumer price index (CPI), food and non-alcoholic beverage consumer price index (FCPI), and real food price level (FPI) reported monthly from January 2017 to December 2020 (2015 = 100). Values displayed represent the average monthly price levels across 160 countries.

Fig. 3 presents the trends in global average FPI, FCPI, and CPI levels between January 2017 and December 2020. Global average CPI and FCPI levels followed approximately parallel paths between January 2017 and December 2019. Accordingly, month-to-month FPI levels remained approximately flat over the same time period. In January 2020 CPI and FCPI levels diverge, with the FCPI rising more quickly than the CPI. By April 2020, the FCPI had risen by approximately 3.7 percentage points from January 2020, while the CPI rose by just 1.6 percentage points over the same time period. The real food price level rose to a high of about 1.6 percentage points above January 2020 levels in April and May 2020, before gradually declining and remaining at approximately 1.2–1.4 percentage points above January 2020 levels between September and December 2020.

#### 3.1.2 Retail price levels and COVID-19 mobility restriction stringency

The number of countries responding to the COVID-19 pandemic with mobility restriction policies increased rapidly during the early months of 2020, as did the overall stringency of these policies (Fig. S4). In our national-level analysis, we sought to investigate the association between retail price levels and stringency in mobility restriction measures that have been introduced during the pandemic. In particular, we were interested in examining whether the relationship between mobility restriction stringency and food retail price levels differed from the relationship between mobility restriction stringency and overall retail price levels for all consumer goods.

**Fig. 4:**
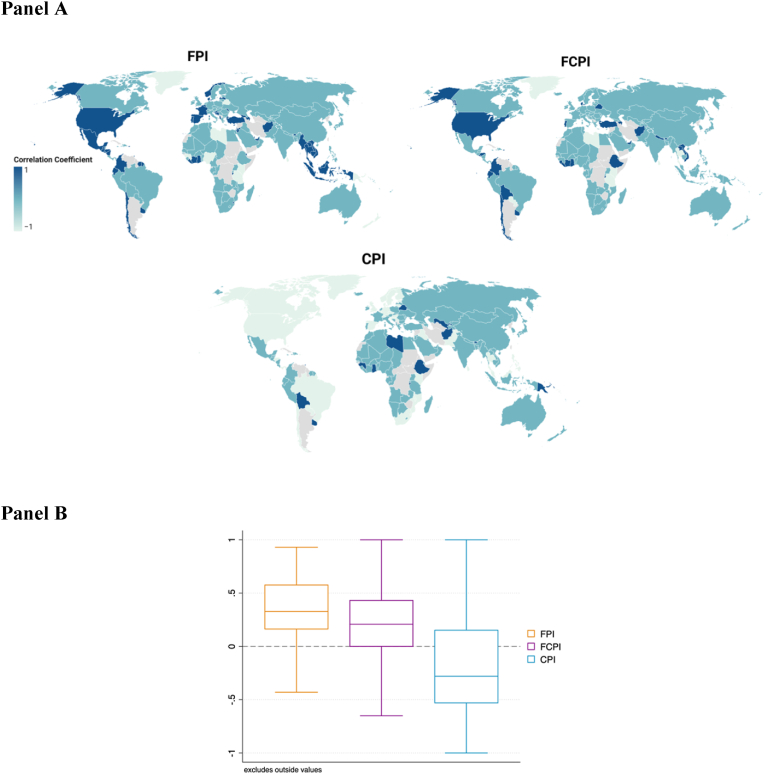
Correlations between mobility restriction stringency and the deviation of each month's price level in 2020 from the average 2017–2019 price level in the same country and month. **Notes:** Panel A displays Spearman's rank correlation coefficients calculated for each country and for each price index (FPI, FCPI, and CPI). Darker shades of blue indicate correlation coefficients closer to positive 1. Panel B displays a box plot to show the spread of correlation coefficients for the FPI, FCPI, and CPI. Monthly national consumer price index data were obtained from the Food and Agriculture Organization of the United Nations on March 17, 2022, and stringency index data were obtained from the Oxford COVID-19 Government Response Tracker on April 28, 2021.

Fig. 4 presents Spearman's rank correlation coefficients, calculated for each individual country, showing the monotonic relationship between mobility restriction stringency and the deviation of each month's FCPI, CPI, or FPI in 2020, relative to that month's 2017–2019 average. The Spearman's rank correlation coefficients for countries were typically positive for the FCPI (mean: 0.19, SD: 0.39) and the FPI (mean: 0.29, SD: 0.42), while correlation coefficients were negative on average for the CPI (mean: 0.17, SD: 0.47).

**Fig. 5:**
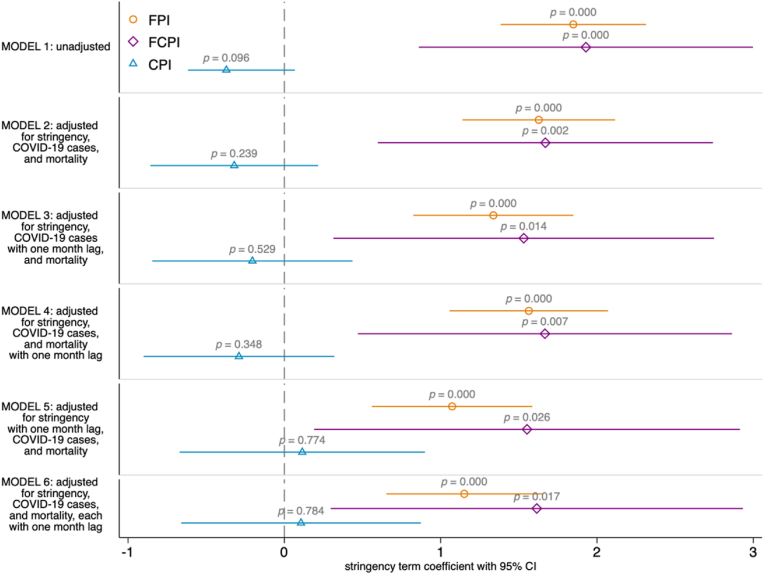
OLS regression estimates of coefficients for mobility restriction stringency **Notes:** Data shown are coefficients, 95 percent confidence intervals, and p-values for the mobility restriction stringency term from six different OLS regressions run for each price index (FPI, FCPI, and CPI). All models include country fixed effects and country time trends, and use robust standard errors clustered at the country level.

To investigate the relationship between mobility restriction stringency and retail price levels more extensively, we used OLS regression with country fixed effects and country specific time trends, and explored potential lag-structures. For each model, the main predictor was COVID-19 mobility restriction stringency and the outcome variable was the deviation of each month's price level (FCPI, CPI, or FPI) in 2020 from that month's average price level during the previous three years (2017–2019). Fig. 5 shows estimates of coefficients and 95 percent confidence intervals for the mobility restriction stringency term from each model, repeated for each price index. Model results are presented in greater detail in Table 1. All regression specifications show a positive and significant association between stringency and FPI deviations during 2020 (β coefficients = 1.073–1.849; 95% confidence intervals [CIs]: 0.560–2.314). These relationships remain positive and significant when country time trends are omitted (Table S5). Interestingly, we also find a positive and significant relationship between stringency with a one-month lag and FPI deviations during 2020. However, this relationship does not remain significant at the five percent significance level when one-month lag terms for COVID-19 cases and mortality are also included in the model (see models 5 and 6 of Table 1). All specifications also indicate a positive and significant association between stringency and FCPI deviations during 2020 (β coefficients = 1.532–1.929; 95% CIs: 0.190–2.998). Mobility restriction stringency was not significantly associated with CPI deviations during 2020 at the five percent significance level.

**Table 1:** Coefficients from OLS regressions for the deviation of each month's price level in 2020 from the average 2017–2019 price level in the same country and month

	(1)	(2)	(3)	(4)	(5)	(6)
**FPI**						
Stringency	1.849*** (0.236)	1.628*** (0.247)	1.337*** (0.259)	1.564*** (0.257)	1.073*** (0.260)	1.151*** (0.252)
Stringency_(t-1)_					0.758*** (0.211)	0.301 (0.249)
ln(C19case + 1)		0.052 (0.033)	0.020 (0.030)	0.052 (0.033)	0.028 (0.032)	0.026 (0.030)
ln(C19case + 1)_(t-1)_			0.145*** (0.031)			0.114*** (0.042)
ln(C19mortality + 1)		−0.006 (0.048)	−0.108** (0.052)	−0.097* (0.049)	−0.003 (0.048)	−0.094* (0.050)
ln(C19mortality + 1)_(t-1)_				0.137*** (0.035)		0.014 (0.046)
Country FE	yes	yes	yes	yes	yes	yes
Country time trends	yes	yes	yes	yes	yes	yes
R-squared (within)	0.554	0.555	0.547	0.540	0.542	0.548
R-squared (between)	0.059	0.061	0.019	0.018	0.018	0.019
R-squared (overall)	0.098	0.100	0.058	0.056	0.056	0.059
N	1837	1837	1657	1657	1657	1657
**FCPI**						
Stringency	1.929*** (0.541)	1.670*** (0.543)	1.532** (0.616)	1.667*** (0.606)	1.552** (0.689)	1.615** (0.668)
Stringency_(t-1)_					0.233 (0.393)	−0.133 (0.418)
ln(C19case + 1)		0.053 (0.054)	0.031 (0.060)	0.050 (0.070)	0.034 (0.063)	0.033 (0.065)
ln(C19case + 1)_(t-1)_			0.085 (0.062)			0.091 (0.075)
ln(C19mortality + 1)		0.012 (0.076)	−0.061 (0.092)	−0.053 (0.089)	−0.001 (0.078)	−0.074 (0.088)
ln(C19mortality + 1)_(t-1)_				0.078 (0.074)		0.011 (0.080)
Country FE	yes	yes	yes	yes	yes	yes
Country time trends	yes	yes	yes	yes	yes	yes
R-squared (within)	0.827	0.828	0.809	0.809	0.809	0.809
R-squared (between)	0.436	0.435	0.437	0.438	0.438	0.437
R-squared (overall)	0.447	0.446	0.437	0.438	0.438	0.437
N	1837	1837	1657	1657	1657	1657
**CPI**						
Stringency	−0.371* (0.222)	−0.321 (0.032)	−0.205 (0.324)	−0.291 (0.309)	0.115 (0.398)	0.107 (0.388)
Stringency_(t-1)_					−0.554** (0.242)	−0.501** (0.242)
ln(C19case + 1)		−0.008 (0.032)	0.011 (0.035)	−0.002 (0.042)	0.005 (0.037)	0.006 (0.039)
ln(C19case + 1)_(t-1)_			−0.056 (0.035)			−0.015 (0.037)
ln(C19mortality + 1)		−0.008 (0.035)	0.021 (0.040)	0.0175 (0.036)	−0.021 (0.037)	−0.011 (0.040)
ln(C19mortality + 1)_(t-1)_				−0.055 (0.040)		0.002 (0.035)
Country FE	yes	yes	yes	yes	yes	yes
Country time trends	yes	yes	yes	yes	yes	yes
R-squared (within)	0.921	0.921	0.910	0.910	0.910	0.910
R-squared (between)	0.338	0.338	0.351	0.350	0.350	0.350
R-squared (overall)	0.362	0.362	0.358	0.358	0.358	0.358
N	1837	1837	1657	1657	1657	1657

**Notes:** Robust standard errors clustered at the country level are in parentheses. ***p < 0.01, **p < 0.05, *p < 0.1.

### 3.2 Market-level analysis

#### 3.2.1 Retail price levels and stay-at-home behavior in 395 markets of 36 countries

**Fig. 6:**
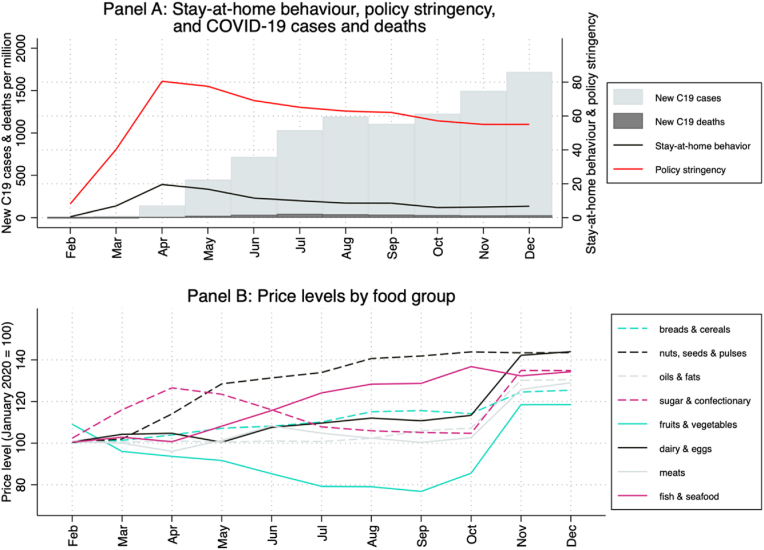
Stay-at-home behavior, mobility restriction stringency, COVID-19 cases and mortality, and retail food price levels across 395 markets in 36 countries, 2020 **Notes:** Panel A shows monthly averages for stay-at-home behaviour (obtained from Google's COVID-19 Community Mobility Trends, https://www.google.com/covid19/mobility), mobility restriction stringency (obtained from the Oxford COVID-19 Government Response Tracker Stringency Index, https://covidtracker.bsg.ox.ac.uk), and new confirmed COVID-19 cases and mortality per million (obtained from Our World in Data's COVID-19 Data Explorer, https://ourworldindata.org/coronavirus) from February to December 2020. Panel B shows monthly average price levels during 2020, by food group, for food items in markets monitored by early warning systems, including the Global Information and Early Warning System (GIEWS) from the Food and Agriculture Organization (FAO) (http://www.fao.org/giews/en/), the Famine Early Warning System Network (FEWS NET) funded by USAID (https://fews.net/), and the Vulnerability Analysis and Mapping (VAM) system from the World Food Programme (WFP) (https://data.humdata.org/dataset/global-wfp-food-prices). Dashed lines indicate less perishable food groups, while solid lines indicate more perishable groups. Values represent monthly levels averaged across 395 markets in 36 countries.

Next, we look in detail at stay-at-home behavior and retail food prices by food group. Panel A of Fig. 6 shows average monthly admin 1-level stay-at-home behavior, national-level mobility restriction stringency, and national-level new confirmed COVID-19 cases and mortality per million between February and December 2020, across 395 markets in 132 admin-1 units of 36 countries included in our market-level analysis. The evolution of stay-at-home behavior roughly follows the trend in mobility restriction stringency, with a Spearman correlation of 0.614 between the two mobility indicators. Both stay-at-home behavior and mobility restriction stringency peak during April 2020 and slowly decline thereafter. Additionally, stay-at-home behavior reflects movement trends for other place categories published by Google (i.e., grocery & pharmacy, transit stations, retail & recreation, parks, and workplaces), with more stay-at-home behavior corresponding to less time spent in other place categories (Fig. S5). Correlations between stay-at-home behavior or mobility restriction stringency and new COVID-19 cases and mortality are weak. The Spearman correlations between mobility restriction stringency and new COVID-19 cases and mortality reach only 0.416 and 0.414, respectively. The Spearman correlations between stay-at-home behavior and new COVID-19 cases and mortality are even weaker, at 0.151 and 0.297, respectively.

Panel B of Fig. 6 shows that within the sample of 395 markets included in our market-level analysis, monthly price levels for most food groups gradually rose throughout the first year of the pandemic, with the most dramatic increases occurring for nuts, seeds, and pulses and dairy and eggs. Fruits and vegetables and meats were the only food groups to show initial drops below pre-pandemic January 2020 baseline levels. However, these decreases were transient, with prices for both food groups returning to and then surpassing baseline levels during subsequent months.

**Fig. 7:**
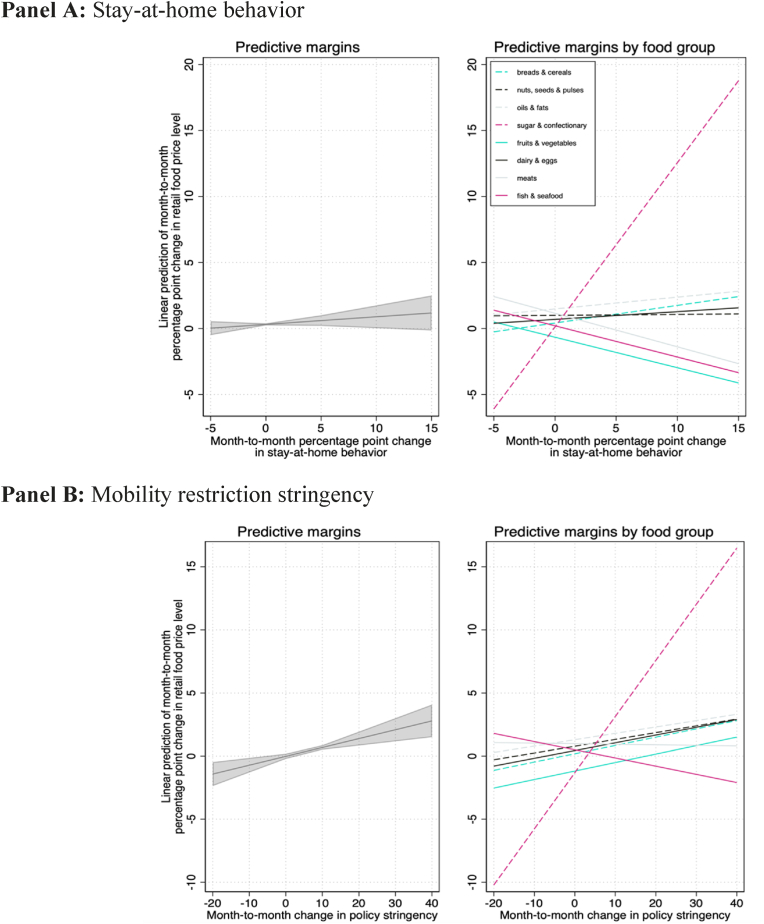
Predictive margins from OLS regression of month-to-month change in stay-at-home behaviour or mobility restriction stringency on month-to-month change in food price level. **Notes:** Data shown are predictive margins from OLS regressions of month-to-month change in stay-at-home behavior (Panel A) or month-to-month change in mobility restriction stringency (Panel B) on month-to-month change in food price level. Visualizations on the left show predictive margins from OLS regressions with all food items pooled together, while visualizations on the right show predictive margins by food group. Dashed lines indicate less perishable food groups, while solid lines indicate more perishable groups. Models control for log(x+1)-transformed COVID-19 case counts and mortality per million per month and include market fixed effects, month fixed effects, and county time trends, and use robust standard errors clustered at the market level. Positive values along the x-axis indicate more time spent at home (Panel A) or higher stringency (Panel B).

In our market-level analysis, we investigated the relationship between month-to-month change in retail food price levels by food group and month-to-month change in stay-at-home behavior using OLS regression with market fixed effects, month fixed effects, and country time trends. Predictive margins from our regressions, controlling for COVID-19 cases and mortality, are shown in panel A of Fig. 7. Detailed model output is presented in Table 2. When all food items are pooled together, there is a positive association between month-to-month change in stay-at-home behavior and month-to-month change in price level, though this relationship is not significant at the five percent significance level (β coefficient = 0.058; 95% CI: 0.035–0.150). Including a food group control in the model and plotting the predictive margins by food group reveals differential trends across groups. Less perishable food groups (breads and cereals, nuts, seeds and pulses, oils and fats, sugar and confectionary), as well as dairy and eggs, show positive associations between month-to-month change in stay-at-home behaviour and month-to-month change in price level. Notable among these is the sugar and confectionary group, which shows a particularly strong and positive association between stay-at-home behaviour and price level change. The three remaining perishable food groups (fruits and vegetables, meats, fish and seafood) show negative associations between month-to-month change in stay-at-home behaviour and month-to-month change in price level.

**Table 2:** Coefficients from OLS regression models of month-to-month change in stay-at-home behaviour or mobility restriction stringency on month-to-month change in food price level

	(1)	(2)	(3)	(4)	(5)	(6)
Stringency				0.065***	0.070***	0.066***
				(0.019)	(0.019)	(0.021)
Stay-at-home behaviour	0.073*	0.058	0.134***			
	(0.041)	(0.047)	(0.051)			
*Food group*						
Breads & cereals			Reference			Reference
Fruits & veg.			−1.077***			−1.370***
			(0.174)			(0.174)
Nut, seed, pulses			0.580**			0.598**
			(0.273)			(0.286)
Dairy & eggs			0.274**			0.253*
			(0.127)			(0.133)
Meats			0.736***			0.799***
			(0.098)			(0.110)
Oils & fats			1.068***			1.113***
			(0.129)			(0.132)
Sugar & confect.			−0.292**			−1.501***
			(0.126)			(0.155)
Fish & seafood			−0.212			0.315
			(0.302)			(0.365)
Food group *mobility indicator						
Breads & cereals			Reference			Reference
Fruits & veg.			−0.364***			0.001
			(0.060)			(0.016)
Nut, seed, pulses			−0.127			−0.012
			(0.081)			(0.017)
Dairy & eggs			−0.075			−0.004
			(0.050)			(0.012)
Meats			−0.388***			−0.070***
			(0.039)			(0.011)
Oils & fats			−0.044			−0.015*
			(0.029)			(0.009)
Sugar & confect.			1.110***			0.379***
			(0.063)			(0.019)
Fish & seafood			−0.370***			−0.131***
			(0.099)			(0.027)
ln(C19case + 1		2.104***	2.096***		2.028***	2.032***
		(0.337)	(0.337)		(0.331)	(0.328)
ln(C19mortality + 1)		−3.135***	−3.027***		−3.146***	−3.137***
		(0.414)	(0.416)		(0.413)	(0.409)
Market FE	yes	yes	yes	yes	yes	yes
Month FE	yes	yes	yes	yes	yes	yes
Country time trends	yes	yes	yes	yes	yes	yes
N	68,167	67,795	67,795	68,167	67,795	67,795
Adjusted R-squared	0.045	0.049	0.069	0.046	0.050	0.065

**Notes:** Robust standard errors clustered at the market level are in parentheses. ***p < 0.01, **p < 0.05, *p < 0.1.

We additionally test the relationship between month-to-month change in retail food price levels by food group and month-to-month change in mobility restriction stringency in the same sample of 36 countries (panel B of Fig. 7). The output from these models generally aligns with our results using stay-at-home behavior. We find a positive and significant association between month-to-month change in stringency and month-to-month change in price level for all food items pooled together (β coefficient = 0.070; 95% CI: 0.033–0.107). When a food group control is included in the model, the directions of association between month-to-month change in stringency and month-to-month change in price level by food group are similar to those using month-to-month change in stay-at-home behavior, with the exception of the fruits and vegetables food group. The direction of association for fruits and vegetables switches from negative to positive when month-to-month change in stringency is used in place of month-to-month change in stay-at-home behavior. This may suggest that mobility restriction stringency and stay-at-home behaviour proxy different sets of COVID-19 impacts to fruit and vegetable supply chains. In our sample, stay-at-home behavior may align more closely with substantial reductions in demand that place downward pressure on prices for perishable foods like fruits and vegetables. A downward shift in demand for fruits and vegetables, corresponding to a higher degree of stay-at-home behavior, would align with a growing number of studies that show reduced purchases of nutrient-rich foods in response to income loss during the pandemic (Ceballos et al., 2021; Hirvonen et al., 2021; Van Hoyweghen et al., 2021). On the other hand, mobility restriction stringency may be a more appropriate proxy of supply chain disruptions and increased supply costs in fruit and vegetable supply chains.

## 4 Discussion

Nutritious diets were already unaffordable for many across the globe even before the COVID-19 pandemic and subsequent food price rises. The UN agencies’ 2020 edition of *The State of Food Security and Nutrition in the World* established that healthy diets, as defined by ten national food-based dietary guidelines from all regions of the world, were unaffordable for some three billion people globally prior to the pandemic (WFP, 2020). This finding is reinforced by a number of recent publications that use least-cost combinations of food items as a metric by which to assess the (un)affordability of diets meeting various requirements (Dizon et al., 2019; Bai et al., 2021a; Hirvonen et al., 2020; Masters et al., 2018). Combined with the other economic impacts of COVID-19 to consumers, such as lost livelihoods and household income losses (Gupta et al., 2021; Laborde et al., 2020a, Laborde et al., 2020b; Maredia et al., 2022), higher food retail price levels during the pandemic further threatened access to affordable and nutritious diets. Accordingly, UN system flagship annual report on *The State of Food Security and Nutrition in the World* estimates that the number of people that could not afford a healthy diet rose to 3.1 billion in 2020 (FAO, IFAD, UNICEF, WFP and WHO, 2022).

### 4.1 Key findings and limitations of the study

Our national-level analysis identifies a positive association between government response stringency in mobility restriction policies and retail food price levels, but not overall inflation in all consumer goods. Among the sample of mostly low- and middle-income countries in our market-level analysis, we find differential trends in the association between stay-at-home behavior and retail food prices, where increased stay-at-home behavior is associated with a rise in the price of non-perishable foods, yet a decrease in price of most perishable foods.

The national-level analysis contributes to our understanding of the impacts of mobility restrictions on retail price levels, though there are some limitations that should be noted. First, expenditure weights used in the calculation of CPI and FCPI data are based on surveys conducted before the COVID-19 outbreak. As such, these expenditure weights do not reflect any shifts in consumer spending habits that occurred during the pandemic. Additionally, the consumer price index cannot be disaggregated to reveal its food item composition or individual food item prices, and it does not provide any indication of subnational variation in price level. To compensate for this, we complement our national-level analysis with a market-level analysis that uses food item price observations from markets monitored by early warning systems, matched to admin 1 level data on stay-at-home behavior. These data allow us to assess relationships between mobility and food price levels by food group, while taking into account subnational variation, but also have important limitations. Item price and stay-at-home behavior data were only available for a smaller sample of 36 countries, compared to 160 countries included in the national-level analysis. Further, food item price data may not reflect the food items and quantities purchased by consumers and these price data cannot be used to produce nationally representative estimates.

### 4.2 Policy implications

Given that mobility restriction policies have been widely applied and changes in stay-at-home behaviour widely observed during the COVID-19 pandemic, our results underscore the urgent need to prioritize measures that improve access to healthy and affordable diets. At the same time, it should be recognized that responses to limit disease spread and severity are critical to minimizing pandemic-related disruptions to retail markets and eventually allowing face-to-face interactions to resume. Applying combinations of policies—i.e., implementing mobility restriction policies alongside other measures such as food assistance programs and protection of consumer incomes—is important to mitigate unwanted spill-over effects from COVID-19 mobility restriction policies into other domains, such as food security and nutrition. Most countries, including all of the countries in this analysis, augmented social protection programs in some way alongside mobility restrictions during the first year of the COVID-19 pandemic. However, only an estimated 20 percent of the world's population were beneficiaries of COVID-related social assistance and average per-capita spending on social assistance was low in lower- and middle-income countries (Gentilini et al., 2021).

The data presented here demonstrate the importance of closely monitoring price levels, not just for commonly traded and stored commodities, but also for foods in retail markets. In recognition of this gap, new work has been conducted to describe and compile publicly available food price index data from national governments and international agencies (Bai et al., 2021b). However, this work notes the need for increased transparency, accessibility, standardization, and timeliness in price index data reporting. Improved availability of disaggregated retail food price data would facilitate future explorations of how retail price levels fluctuate within different food groups or for individual food items. As the COVID-19 pandemic has evolved, it has demanded rapid government responses, which could in turn be informed, calibrated, and improved by real-time and disaggregated data on retail food prices (Adewopo et al., 2021). Improvements to the monitoring and reporting of retail food price data will be key to better understanding the mechanisms that influence retail food price levels, and to supporting better policymaking in response to the COVID-19 crisis, as well as future disease outbreaks.

## CRediT authorship contribution statement

**Jessica Wallingford:** Data curation, Data analysis – original draft, Investigation, Methodology, Visualization, Writing – original draft. **Elena M. Martinez:** Data analysis – review & additional analyses, Writing – review & editing. **William A. Masters:** Resources, Conceptualization, Methodology, Investigation, Funding acquisition, Writing – review & editing.

## Funding

This work was supported by the 10.13039/100000865Bill & Melinda Gates Foundation and UKAid as part of the Food Prices for Nutrition project (INV-016158).

## Declaration of competing interest

The authors declare the following financial interests/personal relationships which may be considered as potential competing interests:

William A. Masters reports financial support and article publishing charges were provided by Bill & Melinda Gates Foundation through the Food Prices for Nutrition Project (INV-016158), with additional financial support from the Foreign Commonwealth & Development Office of the United Kingdom.

## Data Availability

“Replication materials will be posted at the project website, https://sites.tufts.edu/foodpricesfornutrition”
